# Association Study Between *SLC15A4* Polymorphisms and Haplotypes and Systemic Lupus Erythematosus in a Han Chinese Population

**DOI:** 10.1089/gtmb.2015.0289

**Published:** 2016-08-01

**Authors:** Mingwang Zhang, Fangru Chen, Dongmei Zhang, Zhifang Zhai, Fei Hao

**Affiliations:** ^1^ Department of Dermatology, Southwest Hospital, Third Military Medical University, Chongqing, China.; ^2^ Department of Dermatology, Affiliated Hospital of Guilin Medical College, Guilin, China.

## Abstract

*Objective:* The gene *SLC15A4* (solute carrier family 15 [oligopeptide transporter], member 4) has been reported as contributing to the pathogenesis of systemic lupus erythematosus (SLE). We performed a case–control replication study to investigate further the association between single-nucleotide polymorphisms (SNPs) in the *SLC15A4* gene and systemic SLE in a Han Chinese population. *Methods:* In Han Chinese SLE patients and healthy individuals (*n* = 355, 375, respectively), 18 SNPs in the *SLC15A4* gene were genotyped using matrix-assisted laser desorption/ionization time-of-flight mass spectrometry and TaqMan SNP genotyping assays. Analyses of allele frequencies and genotypes using codominant, dominant, and recessive models were conducted, as well as a linkage disequilibrium analysis. *P* values < 0.05 were considered significant. *Results:* Allele frequencies of five of the analyzed SNPs were significantly associated with SLE. Under a codominant model the genotype frequencies of rs3765108 AG and rs7308691 AT were significantly higher in the SLE group than the control group (*p* = 0.019, 0.049, respectively). Under a dominant model the rs1385374 (TT+CT) SNP carried a higher risk of SLE than (CC) (*p* = 0.042). One *SLC15A4* haplotype (TA), which consists of 2 SNPs (rs959989 and rs983492), was associated with SLE (*p* = 0.024). *Conclusion:* Our study determined that five SNPs (rs959989, rs1385374, rs983492, rs12298615, and rs10847697) are associated with SLE. Thus, *SLC15A4* may be important in the pathogenesis of SLE in Han Chinese patients.

## Introduction

Systemic lupus erythematosus (SLE) is a complex autoimmune disease with multiple organ injury. SLE is characterized by aberrant T- and B-cell activation, leading to upregulation of autoantibody production and loss of immunological tolerance to self-nuclear antigens. The etiology of SLE is complex and involves the interaction of genetic, epigenetic, environmental, hormonal, and immunoregulatory factors (Tsokos, 2011). In recent years, the genes causing SLE have been identified, with more than 40 susceptible loci, by using targeted, genome-wide linkage analysis and, especially, genome-wide association studies (GWASs).

*SLC15A4* (solute carrier family 15, member 4), is a proton-coupled histidine and oligopeptide cotransporter that contains 12 membrane-spanning domains. *SLC15A4* is predominantly transcribed in the brain and immune cells, and especially in plasmacytoid dendritic cells (Yamashita *et al.*, 1997; Sasawatari *et al.*, 2011; Nakamura *et al.*, 2014). Through research with *SLC15A4*-mutant or *SLC15A4*-deficient mice, lack of *SLC15A4* has been linked to impaired nucleotide oligomerization domain-1 (NOD1)-, toll-like receptor 7 (TLR7)-, and TLR9-dependent cytokines such as type 1 interferon (IFN), all of which are indispensable to the pathogenesis of SLE (Sasawatari *et al.*, 2011; Baccala *et al.*, 2013; Kobayashi *et al.*, 2014).

Recent GWASs reported that *SLC15A4* is a lupus-associated locus in both the Chinese and Korean populations (Han *et al.*, 2009; Lee *et al.*, 2014). A genotype–phenotype analysis indicated that the single-nucleotide polymorphism (SNP) rs10847697 of the gene has a stronger genetic effect on SLE patients with discoid rash in Han Chinese people (He *et al.*, 2010). Another group showed that the SNPs rs10847697 and rs1385374 of *SLC15A4* were significantly associated with renal involvement in SLE in Caucasian populations (Wang *et al.*, 2013). All of these observations indicate that mutants or polymorphisms of *SLC15A4* have an indispensable role in the pathogenesis of SLE.

To our knowledge, previous studies have focused only on the two SNPs identified from the GWASs. However, the GWASs genotyped hundreds of thousands of tag-SNPs using the Illumina platform, which demands a highly significant *p*-value, and therefore, some SNPs associated with SLE may have been missed.

In the present study, we evaluated associations between *SLC15A4* polymorphisms and SLE in southwestern Han Chinese people through comparisons between 355 SLE patients and 375 healthy individuals. In particular, we focused on the association of alleles and genotypes of 18 SNPs with SLE, and a linkage disequilibrium analysis of these SNPs.

## Materials and Methods

The local regional ethics committee approved the study. All participants provided written informed consent.

### Patients and controls

A case–control study was performed with 355 SLE patients (10 men and 345 women, mean age 32.3 ± 12.5 years) and 375 healthy individuals (control group; 18 men and 357 women, mean age 34.0 ± 7.6 years). All subjects were from the Chongqing, Sichuan, Yunnan, or Guizhou areas of southwestern China and are of Han Chinese ethnicity. Patients were recruited from the Department of Dermatology, Southwestern Hospital of the Third Military Medical University (Chongqing, China) from January 2008 to March 2013. All patients with SLE had fulfilled the revised diagnostic criteria of the American College of Rheumatology (1997) and excluded other systematic or autoimmune diseases (Hochberg, 1997). Blood samples from the control group were collected at the Medical Examination Center of the same hospital during regular health examinations. All of the healthy individuals were free of history or symptoms of SLE or other autoimmune diseases.

### DNA samples and genotyping assays

Venous peripheral blood (2 mL) was collected from each participant with ethylenediaminetetraacetic acid anticoagulant and stored at −80°C until used. Genomic DNA was isolated using a genomic DNA extraction kit (Sangon Biotech) in accordance with the manufacturer's instructions.

Eighteen SNPs were selected based on a tagger SNP selection algorithm using the default settings in Haploview 4.2 or associations with SLE that have been reported in previous studies (Han *et al.*, 2009). In addition, all SNPs came from the international HapMap project databank for the Han Chinese population of Beijing, China, with a minor allele frequency >0.05. For genotyping the 18 SNPs with appropriate methods, 16 of the 18 SNPs were detected by matrix-assisted laser desorption/ionization time-of-flight mass spectrometry (MALDI-TOF MS), with an SNP sequence-specific extension primary added into the polymerase chain reaction (PCR) amplification products (Sangon Biotech). By using a MassARRAY Nanodispenser (Sequenom), the completed genotyping reactions were spotted onto a 384-well SpectroCHIP (Sequenom) and measured using transient (10^−9^ ns) strong laser excitation. Genotype determination was performed in real time with MassARRAY RT software version 3.0.0.4 and analyzed using the MassARRAY Typer software version 3.4 (Sequenom). The genotyping success rate was >95%.

Genotyping of the remaining two SNPs (rs 7965732, rs 3765108) was performed using TaqMan SNP Genotyping Assays on a LightCycler480 real-time PCR system (Roche). Amplification was performed in a 20-μL reaction volume consisting of 10 μL 2× TaqMan Master Mix, 1 μL 20× Primer and TaqMan Probe (FAM VIC) dye mix, 7 μL ddH_2_O, and 2 μL template DNA. The following thermal cycle conditions were used: prerun at 95°C for 4 min; then denaturation at 95°C for 15 s; and an annealing step at 60°C for 1 min in each of 40 cycles.

### Statistical analysis

Data were analyzed with SPSS software for windows (version 17.0; SPSS). The Hardy–Weinberg equilibrium test was applied to examine the genotype frequency of each SNP, using the exact chi-squared test for both the SLE patients and the healthy controls. Linkage disequilibrium blocks of all SNPs were analyzed with Haploview. Conditional logistic regression analysis was applied to evaluate the independent effect of the associated SNPs. The allele, genotype, and haplotype frequency distributions between the SLE patients and healthy individuals were assessed with the chi-squared test and Fisher's exact test. A *post hoc* power analysis was conducted using Power Analysis and Sample Size (PASS) software (NCSS Statistical Software). Estimated odds ratios (ORs) and 95% confidence intervals (CIs) were also calculated based on logistic regression. All the tests were two sided and *p*-values <0.05 were regarded as significant.

## Results

### Allele frequency distribution of *SLC15A4* polymorphism

The ages and genders of the patient and control groups were statistically similar. All polymorphic frequencies were confirmed to be in the Hardy–Weinberg equilibrium in both groups.

The distribution of allele frequencies of 5 of the 18 SNPs from *SLC15A4* was significantly different between the groups (Table 1). Specifically, the following SNPs were associated with an increased risk of SLE: rs959989 T (OR 1.317, 95% CI 1.027–1.689), rs1385374 T (OR 1.332, 95% CI 1.039–1.707), and rs983492 T (OR 1.295, 95% CI 1.001–1.675). The *post hoc* calculated statistical power of these SNPs (rs959989 T, rs1385374 T, and rs983492 T) was 58.5%, 62.0%, and 51.1%, respectively.

**Table 1.** Distribution of Allele Frequencies in Single-Nucleotide Polymorphisms of *SLC15A4* in Systemic Lupus Erythematosus Patients and Controls

		*SLE (*n = *355)*		*Control (*n = 375*)*			
*SNP*	*Allele*	*Count*	*%*	*Count*	*%*	*OR (95% CI)*	P
rs7965732	A	642	90.42	671	89.47	Reference	
	T	68	9.57	79	10.53	0.9 (0.639, 1.266)	0.544
rs3765108	A	479	67.46	512	68.27	Reference	
	G	231	32.54	238	31.73	1.037 (0.833, 1.292)	0.743
rs7308691	A	233	32.91	237	31.77	Reference	
	T	475	67.09	509	68.23	0.949 (0.762, 1.183)	0.642
rs7302634	C	307	43.48	322	42.93	Reference	
	T	399	56.52	428	57.07	0.978 (0.795, 1.203)	0.832
rs9738216	C	307	43.36	321	42.91	Reference	
	T	401	56.64	427	57.09	0.982 (0.798, 1.208)	0.863
rs2291350	C	309	43.64	322	42.93	Reference	
	T	399	56.36	428	57.07	0.971 (0.790, 1.195)	0.784
rs11059915	G	546	76.9	562	74.93	Reference	
	T	164	23.1	188	25.07	0.898 (0.706, 1.142)	0.38
rs12298615	A	169	31.35	147	19.6	Reference	
	G	539	68.65	603	80.4	0.778 (0.606, 0.998)	0.048^a^
rs4760592	C	401	56.48	429	57.35	Reference	
	G	309	43.52	319	42.65	1.036 (0.842, 1.275)	0.736
rs959989	A	537	75.63	601	80.35	Reference	
	T	173	24.37	147	19.65	1.317 (1.027, 1.689)	0.03^a^
rs959987	C	578	81.64	580	77.54	Reference	
	T	130	18.36	168	22.46	0.776 (0.601, 1.003)	0.053
rs7311875	A	537	76.06	559	74.53	Reference	
	G	169	23.94	191	25.47	0.921 (0.726, 1.169)	0.499
rs11059925	A	558	80.64	593	79.07	Reference	
	C	134	19.36	157	20.93	0.907 (0.701, 1.174)	0.458
rs10847697	A	173	24.37	149	19.87	Reference	
	G	537	75.63	601	80.13	0.770 (0.600, 0.986)	0.038^a^
rs1385374	C	536	75.49	603	80.4	Reference	
	T	174	24.51	147	19.6	1.332 (1.039, 1.707)	0.024^a^
rs10847699	C	172	24.36	192	25.67	Reference	
	T	534	75.64	556	74.33	1.072 (0.845, 1.360)	0.566
rs983492	C	127	18.46	170	22.67	Reference	
	T	561	81.54	580	77.33	1.295 (1.001, 1.675)	0.049^a^
rs1552336	C	637	89.72	657	87.83	Reference	
	T	73	10.28	91	12.17	0.827 (0.597, 1.147)	0.256

^a^ Significant association (*p* < 0.05).

CI, confidence interval; OR, odds ratio; SLE, systemic lupus erythematosus; SNP, single-nucleotide polymorphism.

Associated with a decreased risk of SLE were rs12298615 G (OR 0.778, 95% CI 0.606–0.998) and rs10847697 G (OR 0.770, 95% CI 0.600–0.986). We performed a *post hoc* power analysis and found that the statistical power of rs12298615 G was 50.1% and that of rs10847697 G was 54.3%, with a 5% type-1 error rate.

### Genotype frequency distribution of *SLC15A4* polymorphism

For better understanding the effect of the SNP genotype on the risk of SLE, the genotype distributions of the SLE and control groups were compared using codominant, dominant, and recessive models (Tables 2–4, respectively). The results of the codominant model (Table 2) indicate that in both the SLE and control groups, the frequency distribution of the AG genotype of rs3765108 was significantly higher than that of the AA genotype (*p* = 0.019, OR 1.447, 95% CI 1.063–1.970), and the frequency distribution of the AT genotype of rs7308691 was significantly higher than that of the AA genotype (*p* = 0.049, OR 1.645, 95% CI 1.000–2.705).

**Table 2.** Codominant Analysis of Single-Nucleotide Polymorphisms in *SLC15A4* in Systemic Lupus Erythematosus Patients and Controls

		*SLE (*n = 355*)*		*Control (*n = 375*)*			
*SNP*	*Genotype*	*Count*	*%*	*Count*	*%*	*OR (95% CI)*	P
rs7965732	AA	290	81.69	302	80.53	Reference	
	TT	3	0.85	6	1.6	0.521 (0.129, 2.101)	0.551
	AT	62	17.46	67	17.87	0.964 (0.658, 1.411)	0.849
rs3765108	AA	154	43.38	185	49.33	Reference	
	GG	30	8.45	48	12.8	0.751 (0.454, 1.243)	0.264
	AG	171	48.17	142	37.87	1.447 (1.063, 1.970)	0.019^a^
rs7308691	AA	33	9.32	49	13.14	Reference	
	TT	167	47.18	185	49.6	1.340 (0.822, 2.185)	0.239
	AT	154	43.5	139	37.27	1.645 (1.000, 2.705)	0.049^a^
rs7302634	CC	66	18.7	76	20.27	Reference	
	TT	112	31.73	129	34.4	1.000 (0.660, 1.515)	0.999
	CT	175	49.58	170	45.33	1.185 (0.801, 1.753)	0.394
rs9738216	CC	66	18.64	76	20.32	Reference	
	TT	113	31.92	129	34.49	1.009 (0.666, 1.528)	0.967
	CT	175	49.44	169	45.19	1.192 (0.806, 1.764)	0.378
rs2291350	CC	67	18.93	76	20.27	Reference	
	TT	112	31.64	129	34.4	0.985 (0.651, 1.491)	0.942
	CT	175	49.44	170	45.33	1.168 (0.790, 1.725)	0.436
rs11059915	GG	205	57.75	212	56.53	Reference	
	TT	14	3.94	25	6.67	0.579 (0.293, 1.145)	0.113
	GT	136	38.31	138	36.8	1.019 (0.751, 1.382)	0.903
rs12298615	AA	25	7.06	17	45.33	Reference	
	GG	210	59.32	245	65.33	0.583 (0.306, 1.109)	0.097
	AG	119	33.62	113	30.13	0.716 (0.367, 1.396)	0.326
rs4760592	CC	112	31.55	130	34.76	Reference	
	GG	66	18.59	75	20.05	1.021 (0.674, 1.549)	0.92
	CG	177	49.86	169	45.19	1.216 (0.875, 1.689)	0.245
rs959989	AA	208	48.59	244	65.24	Reference	
	TT	26	7.32	17	4.55	1.794 (0.947, 3.398)	0.07
	AT	121	34.08	113	30.22	1.256 (0.916, 1.723)	0.157
rs959987	CC	237	66.95	228	60.96	Reference	
	TT	13	3.67	22	5.88	0.568 (0.280, 1.156)	0.115
	CT	104	29.38	124	33.16	0.807 (0.587, 1.109)	0.185
rs7311875	AA	200	56.66	211	56.27	Reference	
	GG	16	4.53	27	7.2	0.625 (0.327, 1.195)	0.152
	AG	137	38.81	137	36.53	1.055 (0.777, 1.432)	0.731
rs11059925	AA	221	63.87	234	62.4	Reference	
	CC	9	2.6	16	4.27	0.596 (0.258, 1.376)	0.221
	AC	116	33.53	125	33.33	0.983 (0.719, 1.343)	0.912
rs10847697	AA	26	7.32	18	4.8	Reference	
	GG	208	58.59	244	65.07	0.590 (0.315, 1.107)	0.097
	AG	121	34.08	113	30.13	0.741 (0.386, 1.425)	0.368
rs1385374	CC	207	58.31	246	65.6	Reference	
	TT	26	7.32	18	4.8	1.717 (0915, 3.219)	0.089
	CT	122	34.37	111	29.6	1.306 (0.952, 1.793)	0.098
rs10847699	CC	17	4.82	28	7.49	Reference	
	TT	198	56.09	210	56.15	1.553 (0.824, 2.925)	0.17
	CT	138	39.09	136	36.36	1.671 (0.875, 3.193)	0.117
rs983492	CC	13	3.78	23	6.13	Reference	
	TT	230	66.86	228	60.8	1.785 (0.882, 3.610)	0.103
	CT	101	29.36	124	33.07	1.441 (0.695, 2.988)	0.324
rs1552336	CC	287	80.85	287	76.74	Reference	
	TT	5	1.41	4	1.07	1.250 (0.332, 4.702)	1
	CT	63	17.75	83	22.2	0.759 (0.526, 2.095)	0.139

^a^ Significant association (*p* < 0.05).

**Table 3.** Dominant Analysis of Single-Nucleotide Polymorphisms in *SLC15A4* in Systemic Lupus Erythematosus Patients and Controls

		*SLE (*n = 355*)*		*Control (*n = 375*)*			
*SNP*	*Genotype*	*Count*	*%*	*Count*	*%*	*OR (95% CI)*	P
rs7965732	TT+AT	65	18.31	73	19.47	0.927 (0.640, 1.344)	0.69
	AA	290	81.69	302	80.53	Reference	
rs3765108	GG+AG	201	56.62	190	50.67	1.271 (0.949, 1.701)	0.107
	AA	154	43.38	185	49.33	Reference	
rs7308691	TT+AT	321	90.68	324	86.86	1.471 (0.922, 2.348)	0.104
	AA	33	9.32	49	13.14	Reference	
rs7302634	TT+CT	287	81.3	299	79.73	1.105 (0.765, 1.596)	0.593
	CC	66	18.7	76	20.27	Reference	
rs9738216	TT+CT	288	81.36	298	79.68	1.113 (0.771, 1.607)	0.568
	CC	66	18.64	76	20.32	Reference	
rs2291350	TT+CT	287	81.07	299	79.73	1.089 (0.755, 1.570)	0.649
	CC	67	18.93	76	20.27	Reference	
rs11059915	TT+GT	150	42.25	163	43.47	0.952 (0.710, 1.276)	0.741
	GG	205	57.75	212	56.53	Reference	
rs12298615	GG+AG	329	92.94	358	95.47	0.625 (0.331, 1.178)	0.143
	AA	25	7.06	17	4.53	Reference	
rs4760592	GG+CG	243	68.45	244	65.24	1.156 (0.849, 1.574)	0.358
	CC	112	31.55	130	34.76	Reference	
rs959989	TT+AT	147	41.41	130	34.76	1.326 (0.983, 1.790)	0.065
	AA	208	58.59	244	65.24	Reference	
rs959987	TT+CT	117	33.05	146	39.04	0.771 (0.569, 1.044)	0.093
	CC	237	66.95	228	60.96	Reference	
rs7311875	GG+AG	153	43.34	164	43.73	0.984 (0.734, 1.320)	0.915
	AA	200	56.66	211	56.27	Reference	
rs11059925	CC+AC	125	36.13	141	37.6	0.939 (0.693, 1.271)	0.682
	AA	221	63.87	234	62.4	Reference	
rs10847697	GG+AG	329	92.68	357	95.2	0.638 (0.343, 1.185)	0.152
	AA	26	7.32	18	4.8	Reference	
rs1385374	TT+CT	148	41.69	129	34.4	1.1363 (1.010, 1.840)	0.042^a^
	CC	207	58.31	246	65.6	Reference	
rs10847699	TT+CT	336	95.18	346	92.51	1.599 (0.860, 2.976)	0.135
	CC	17	4.82	28	7.49	Reference	
rs983492	TT+CT	331	96.22	352	93.87	1.664 (0.829, 3.338)	0.148
	CC	13	3.78	23	6.13	Reference	
rs1552336	TT+CT	68	19.15	87	23.26	0.782 (0.547, 1.117)	0.176
	CC	287	80.85	287	76.74	Reference	

^a^ Significant association (*p* < 0.05).

**Table 4.** Recessive Analysis of Single-Nucleotide Polymorphisms in *SLC15A4* in Systemic Lupus Erythematosus Patients and Controls

		*SLE (*n = 355*)*		*Control (*n = 375*)*			
*SNP*	*Genotype*	*Count*	*%*	*Count*	*%*	*OR (95% CI)*	p
rs7965732	TT	3	0.85	6	1.6	0.524 (1.3, 2.112)	0.556
	AA+AT	352	99.15	369	98.4	Reference	
rs3765108	GG	30	8.45	48	12.8	0.629 (0.389, 1.018)	0.057
	AA+AG	325	91.55	327	82.7	Reference	
rs7308691	TT	167	47.18	185	49.6	0.908 (0.678, 1.214)	0.514
	AA+AT	187	52.82	188	50.4	Reference	
rs7302634	TT	112	31.73	129	34.4	0.886 (0.65, 1.207)	0.444
	CC+CT	241	68.27	246	65.6	Reference	
rs9738216	TT	113	31.92	129	34.49	0.891 (0.654, 1.213)	0.462
	CC+CT	241	68.08	245	65.51	Reference	
rs2291350	TT	112	31.64	129	34.4	0.883 (0.648, 1.202)	0.428
	CC+CT	242	68.36	246	65.6	Reference	
rs11059915	TT	14	3.94	25	6.67	0.575 (0.294, 1.124)	0.102
	GG+GT	341	96.06	350	93.33	Reference	
rs12298615	GG	210	59.32	245	65.33	0.774 (0.573, 1.045)	0.094
	AA+AG	144	40.68	130	34.66	Reference	
rs4760592	GG	66	18.59	75	20.05	0.910 (0.630, 1.316)	0.617
	CC+CG	289	81.41	299	79.95	Reference	
rs959989	TT	26	7.32	17	4.55	1.660 (0.884, 3.114)	0.111
	AA+AT	329	92.68	357	95.45	Reference	
rs959987	TT	13	3.67	22	5.88	0.610 (0.302, 1.230)	0.167
	CC+CT	341	96.33	352	94.12	Reference	
rs7311875	GG	16	4.53	27	7.2	0.612 (0.324, 1.156)	0.127
	AA+AG	337	95.47	348	92.8	Reference	
rs11059925	CC	9	2.6	16	4.27	0.599 (0.261, 1.374)	0.222
	AA+AC	337	97.4	359	95.73	Reference	
rs10847697	GG	208	58.59	244	65.07	0.760 (0.563, 1.025)	0.072
	AA+AG	147	41.41	131	34.93	Reference	
rs1385374	TT	26	7.32	18	4.8	1.567 (0.844, 2.912)	0.152
	CC+CT	329	92.68	357	95.2	Reference	
rs10847699	TT	198	56.09	210	56.15	0.998 (0.744, 1.337)	0.987
	CC+CT	155	43.91	164	43.85	Reference	
rs983492	TT	230	66.86	228	60.8	1.301 (0.958, 1.766)	0.091
	CC+CT	114	33.14	147	39.2	Reference	
rs1552336	TT	5	1.41	4	1.07	1.321 (0.352, 4.961)	0.937
	CC+CT	350	98.59	370	98.93	Reference	

According to the analysis using the dominate model (Table 3), only one SNP (rs1385374, TT+CT cf.CC, *p* = 0.042, OR 1.1363, 95% CI 1.010–1.840) was significantly associated with increased risk of SLE. According to the analysis results of the recessive model, there were no significant differences among the 18 SNP genotypes (Table 4).

### Linkage disequilibrium analysis

To confirm that the five detected SNPs (rs959989, rs1385374, rs983492, rs12298615, and rs10847697) are independent, we performed a conditional analysis (Table 5). However, the analysis showed complete abrogation of genetic associations among the five SNPs. This may be due to the relatively high *p-*value before the analysis, due to the small sample size. We found that the association of rs959989 and rs983492 was slightly attenuated, while associations among rs12298615, rs10847697, and rs138537 were markedly attenuated when adjusted for each other (Table 5). In addition, using Haploview, the four SNPs rs12298615, rs959989, rs10847697, and rs138537 are in near absolute linkage disequilibrium (r^2^ > 0.98). Thus, we chose rs959989 from the four SNPs and rs983492 to perform a linkage disequilibrium analysis. Future studies with a large sample are warranted. We also note that other variants not assessed in this study are possible and might contribute to this effect.

**Table 5.** Conditional Logistic Regression Analysis of *SLC15A4* Single-Nucleotide Polymorphisms Associated with Systemic Lupus Erythematosus

		p*_adjusted_ when conditioned on:*				
	p^a^	*rs12298165*	*rs959989*	*rs10847697*	*rs1385374*	*rs983492*
rs12298165	0.056	N/A	0.738	0.874	0.355	0.129
rs959989	0.036	0.146	N/A	0.109	0.136	0.101
rs10847697	0.045	0.895	0.99	N/A	0.99	0.124
rs1385374	0.029	0.222	0.313	0.99	N/A	0.085
rs983492	0.055	0.16	0.155	0.155	0.172	N/A

^a^ *p-*Value, calculated by logistic regression analysis under the additive model.

N/A, not applicable.

**FIG. 1.**
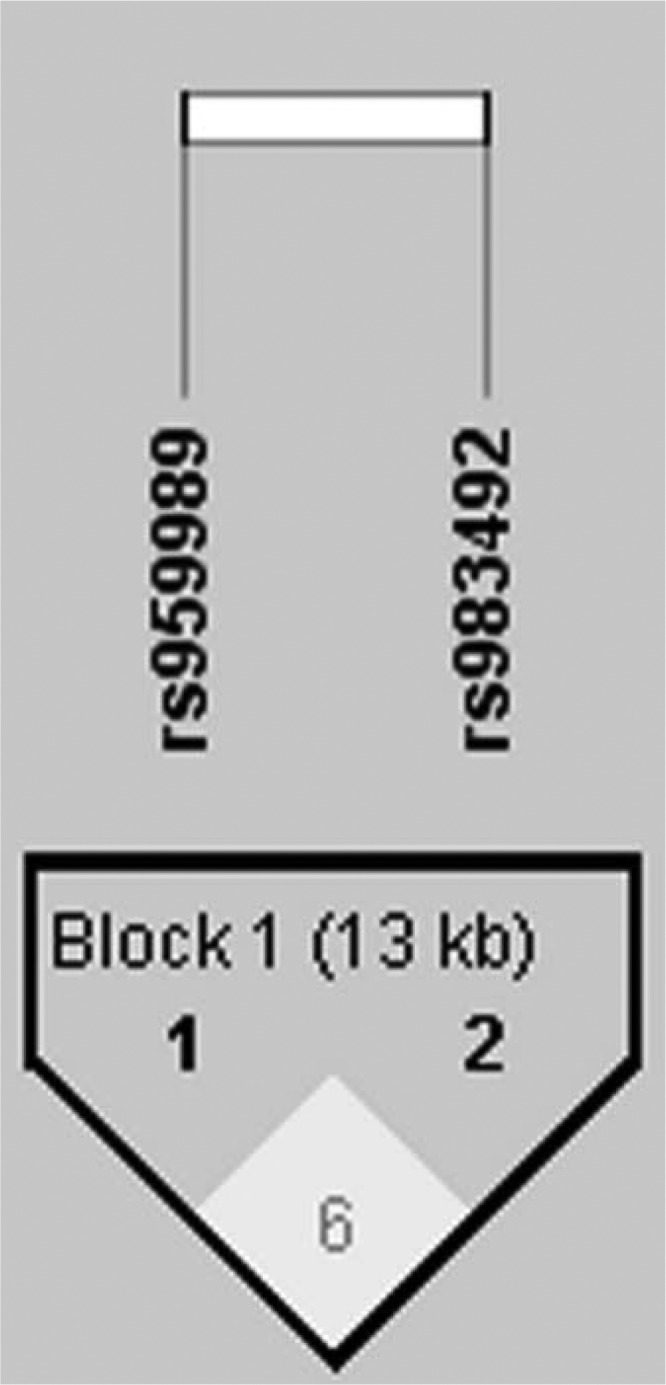
Analysis of linkage disequilibrium of two single-nucleotide polymorphisms in *SLC15A4* in systemic lupus erythematosus patients and controls. The haplotype block structure was analyzed using Haploview software, version 4.2. Each box gives the coefficient of determination (*r*^2^), and the *r*^2^ color scheme is as follows: *white* (*r*^2^ = 0), *gray* (0 < *r*^2^ < 1), *black* (*r*^2^ = 1).

Next, we performed linkage disequilibrium with the two SNPs rs959989 and rs983492 by using Haploview (Fig. 1). Table 6 shows the frequencies of different haplotypes and the results from the analysis of the association with SLE. The prevalence of the haplotype TA was significantly higher in the SLE patients than in the control group (*p* = 0.024, OR 1.333, 95% CI 1.039–1.125).

**Table 6.** Haplotype Analysis in Systemic Lupus Erythematosus Patients and Controls

*Haplotype*	*Total (%)*	*Case (%)*	*Control (%)*	p	*OR (95% CI)*
AT	840 (57.5)	406 (57.2)	434 (57.9)	0.792	0.972 (0.790–1.197)
TA	318 (21.8)	173 (24.4)	146 (19.5)	0.024^a^	1.333 (1.039–1.125)
AC	299 (20.5)	131 (18.5)	168 (22.4)	0.062	0.784 (0.607–1.012)

^a^ Significant association (*p* < 0.05).

## Discussion

In this study, we investigated 18 SNPs in the *SLC15A4* gene with a case–control study and found that five SNPs (rs959989, rs1385374, rs983492, rs12298615, and rs10847697) were associated with SLE in this Han Chinese population. Using a codominant model for analysis, we found that the frequencies of the rs3765108 AG genotype and the rs7308691 AT genotype were significantly higher in the SLE patients, suggesting that these SNPs may be a risk factor for SLE. Moreover, by using the dominant model, the results suggest that rs1385374 (TT+CT cf. CC) was associated with increased risk of SLE. In addition, one *SLC15A4* haplotype was also associated with SLE.

SLE is a systemic autoimmune disease accompanied by auto-antibodies, including anti-DNA and anti-small nuclear ribonucleoprotein (snRNP) antibodies, which lead to multiple organ damage (Liu and Davidson, 2012). Nucleic acid binding receptors such as TLR7 and TLR9 are crucial in SLE, promoting the production of IFN-1 through plasmacytoid dendritic cells and activating autoreactive B cells (Cao *et al.*, 2008; Green and Marshak-Rothstein, 2011). Moreover, TLR7 and IFN-α can act together and induce the switching of B cells to IgG2a/c during influenza infection (Marshak-Rothstein, 2006; Heer *et al.*, 2007). On the contrary, *SLC15A4* was found to be required for the production of antibodies in B cells in a mouse model of lupus, and the lack of *SLC15A4* in B cells led to reduced production of IgG2a and IgG2c auto-antibodies such as anti-snRNP and anti-DNA antibodies (Kobayashi *et al.*, 2014). Interestingly, Dosenovic *et al.* (Dosenovic *et al.*, 2015) demonstrated that a functional *SLC15A4* was required for effective antibody isotype switching to IgG2c, in response to TLR9 stimulation.

Previous studies (Lee *et al.*, 2009; Blasius *et al.*, 2010; Sasawatari *et al.*, 2011; Baccala *et al.*, 2013) reported that *SLC15A4* has a role in maintaining the appropriate endosomal pH and is necessary for the innate immune response triggered by NOD1, NOD2, TLR7, and TLR9. In a feeble mouse model, defective *SLC15A4* led to chronic viral infection by reducing the function of plasmacytoid dendritic cells (Blasius *et al.*, 2012). Previous studies showed that synonymous mutants can affect splicing events, messenger RNA stability, microRNA binding, nucleosome formation, and genes' translational efficiency, sometimes even causing disorders (Chamary *et al.*, 2006; Plotkin and Kudla, 2011; Waldman *et al.*, 2011). In our study, we found a significantly lower risk of SLE associated with the SNP rs10847697, a synonymous polymorphism that has been reported associated with discoid rash or renal disorder (He *et al.*, 2010; Wang *et al.*, 2013). Therefore, this will give us a new research direction to investigate the influence of rs10847697 on the function of *SLC15A4*. The others four SNPs, however, are all located in the intron region and it is hard to investigate their functional studies.

The first GWAS regarding *SLC15A4* showed that it is closely associated with type 2 diabetes in Japan (Takeuchi *et al.*, 2008). Subsequently, another GWAS by Han *et al.* reported that *SLC15A4* is a new susceptibility gene for SLE and identified two SNPs (rs10847697, rs1385374) associated with SLE in a Chinese population. This is in agreement with our results. In addition, several other research projects revealed that many clinical features were associated with the two SNPs (He *et al.*, 2010; Wang *et al.*, 2013; Zuo *et al.*, 2014).

Interestingly, a recent study identified new risk loci associated with SLE, using high-density genotyping based on the Immunochip SNP microarray, in Asian ancestry (Sun *et al.*, 2016). In their research, five SNPs (rs959989, rs1385374, rs983492, rs12298615, and rs10847697) were detected and are generally consistent with our results, except rs983492. We speculate that this difference may be due to different detection methods and racial constitution. Further studies with larger sample sizes are needed to confirm this hypothesis.

The present study is limited in that the *p-*values reflected much less significance compared with previous studies, and no significant difference was indicated after the Bonferroni correction or through the Benjamini–Hochberg method. This may be due to the relatively small sample size of our study (hundreds), compared with previous studies (thousands), which limited statistical power. It also must be noted that patients of other ethnicities may have greater differences in allele frequencies (Mori *et al.*, 2005) than our Han Chinese population. Therefore, our results may not be applicable to other populations. Functional studies of the identified SNPs are warranted, and we are planning a future investigation of the expression and 3′-untranslated region of the gene using microRNAs *in vitro*.

The pathogenesis of SLE is believed to involve the combined effects of a large number of minor genes, and one of these is *SLC15A4*. Our study provides clues that help elucidate the role of the *SLC15A4* gene in the pathogenesis of SLE in Han Chinese people. More comprehensive research that includes populations of different ethnicities is warranted. Further studies should focus on the physiological functions of the mutated loci that occur with significantly different frequencies between SLE and healthy individuals, as well as the influence of SNPs in *SLC15A4* on SLE in the Han Chinese population.
